# Patient-specific osteoarticular reconstruction of the proximal tibia for giant cell tumor using a 3D-printed trabecular implant and hinged knee arthroplasty: a case report

**DOI:** 10.3389/fmed.2026.1868738

**Published:** 2026-09-15

**Authors:** Mauricio Olmedo-López, Mauro Damian Belzino, Jorge Pérez-Soliz, Jorge Vasconez-Gonzalez, Fanny K. Tupiza-Luna, Galo S. Guerrero-Castillo, Juan S. Izquierdo-Condoy, Esteban Ortiz-Prado

**Affiliations:** 1 Orthopedic Surgery Resident, Hospital de Clínicas José de San Martín, University of Buenos Aires, Argentina; 2 Specialist in Orthopedic Surgery, Specialist in Orthopedic Oncology, Staff Physician, Hospital de Clínicas José de San Martín, University of Buenos Aires, Argentina; 3 Specialist in Orthopedic Surgery, Foot and Ankle Fellowship, Trauma Center, Hospital de Clínicas José de San Martín, University of Buenos Aires, Argentina; 4 One Health Research Group, Universidad de Las Américas, Quito, Ecuador; 5 Departamento de Traumatologia y Ortopedia, Hospital Pablo Arturo Suarez, Quito, Ecuador

**Keywords:** arthroplasty, case report, giant cell tumor of bone, knee, knee prosthesis, osseointegration, replacement, three-dimensional printing

## Abstract

**Background:**

Giant cell tumor of bone (GCTB) is a benign but locally aggressive primary bone tumor that frequently affects the epiphyseal–metaphyseal region of long bones, particularly around the knee. In rare cases, it may undergo malignant trans-formation or metastasize.

**Case presentation:**

We report the case of a 62-year-old woman with a 5-year history of left knee pain, progressive deformity, and functional limitation. Imaging studies and biopsy confirmed a giant cell tumor of the proximal tibia with extensive epiphyseal involvement and articular collapse. The patient underwent oncologic resection using patient-specific cutting guides, followed by reconstruction with a custom 3D-printed porous trabecular Ti6Al4V-ELI implant manufactured by selective laser melting on a Renishaw AM400 system, combined with hinged total knee arthroplasty. The extensor mechanism was reinserted through dedicated fixation slots, and soft-tissue coverage was achieved with a medial gastrocnemius flap and polypropylene mesh. At 6 months, radiographs showed satisfactory alignment and early structural integration, with no evidence of local recurrence or implant loosening. Clinically, the patient achieved full weight-bearing and an excellent functional outcome.

**Conclusion:**

This case suggests that patient-specific reconstruction combining virtual planning, 3D printing, and porous trabecular design may be a feasible limb-salvage option for selected proximal tibial GCTB with major osteoarticular destruction.

## 1 Introduction

Giant cell tumor of bone (GCTB) is a benign but locally aggressive primary bone neoplasm characterized by osteolysis, cortical thinning, and a substantial risk of local recurrence (1). It most commonly arises in the epiphyseal–metaphyseal region of long bones, particularly around the knee, with the distal femur and proximal tibia among the most frequently affected sites (2, 3). Although GCTB usually follows a benign course, malignant transformation may occasionally occur, and pulmonary metastases have been reported in 1% to 9% of patients (4, 5).

GCTB accounts for approximately 20% of benign skeletal tumors and 5% of all primary bone tumors (4, 5). Approximately 80% of cases occur between 20 and 50 years of age, 13% in patients older than 50 years, and fewer than 3% in children younger than 14 years (4). A female predominance has been described, with a reported female-to-male ratio of 1.3–1.5:1 and a higher incidence in Asian populations than in Western populations (1).

The proximal tibia deserves particular attention because tumors arising in this location challenge both oncologic control and functional preservation. Resection may compromise the articular surface, extensor mechanism, joint alignment, and periarticular soft tissues, making reconstruction especially demanding (6). Although the distal femur is the most common site for GCTB, the proximal tibia accounts for approximately 18% of cases and is associated with important reconstructive limitations when substantial epiphyseal destruction is present (7).

Currently, three-dimensional (3D) printing has demonstrated significant potential in medical applications, enabling surgeons to improve operative precision through the creation of patient-specific anatomical models and even the development of customized implants (8). Furthermore, the combination of this technology with advanced image segmentation methods and CAD software has enabled more accurate replication of patient anatomy (9). 3D printing also allows for the development of patient-specific surgical instruments, which contributes to greater precision in resection, proper alignment of the mechanical axis, as well as improved cost-effectiveness and time efficiency (10). It has been reported that customized implants improve implant positioning and promote better anatomical reconstruction (8). On the other hand, porous implants have been observed to provide improved biomechanical functionality in the tibial plateau (11). In their review analyzing 46 studies, Safavi et al. identified three porous design strategies: uniform, graded, and optimized. They also noted that controlled porous designs are promising for reducing the stress shielding effect (12).

In lesions with extensive subchondral collapse or major structural compromise, intralesional treatment may be insufficient to ensure durable joint preservation. In such settings, wide resection followed by biologically and mechanically robust reconstruction may represent a reasonable limb-salvage strategy. For this reason, we present a case of proximal tibial GCTB treated with oncologic resection and patient-specific osteoarticular reconstruction using a custom 3D-printed porous trabecular implant combined with a hinged knee arthroplasty. Additionally this case stands out because the trabecular metal implant structure was specifically designed to completely replace the proximal end of the tibia and to adequately support a constrained joint prosthesis without loosening. The advantage of the design lies in its stability (press-fit fixation and screw augmentation) and in the three pairs of eyelets developed for reattachment of the extensor mechanism using high-strength sutures. Furthermore, the articular surface of the implant allows for the placement of “steps” to adjust the size of the prosthesis.

## 2 Case report

For the preparation and presentation of this case report, the CARE guidelines for clinical case reports were followed in order to ensure accuracy and transparency.

A 62-year-old woman with no prior medical, surgical, or trauma history presented with a 5-year history of left knee pain of mechanical characteristics, associated with progressive deformity of the medial aspect of the knee and increasing functional limitation. Physical examination revealed a hard, non-tender mass in the medial region of the knee. Active flexion and extension were limited by pain. No distal neurovascular compromise was observed.

Although the pain began earlier, the patient had presented with the deformity and the mass in her knee since 2023, when she began undergoing evaluation, as demonstrated by the initial radiographs showing an expansile lytic lesion involving the proximal tibia, with poorly defined margins, cortical thinning, and progressive articular collapse on subsequent studies (Figure 1).

**Figure 1:**
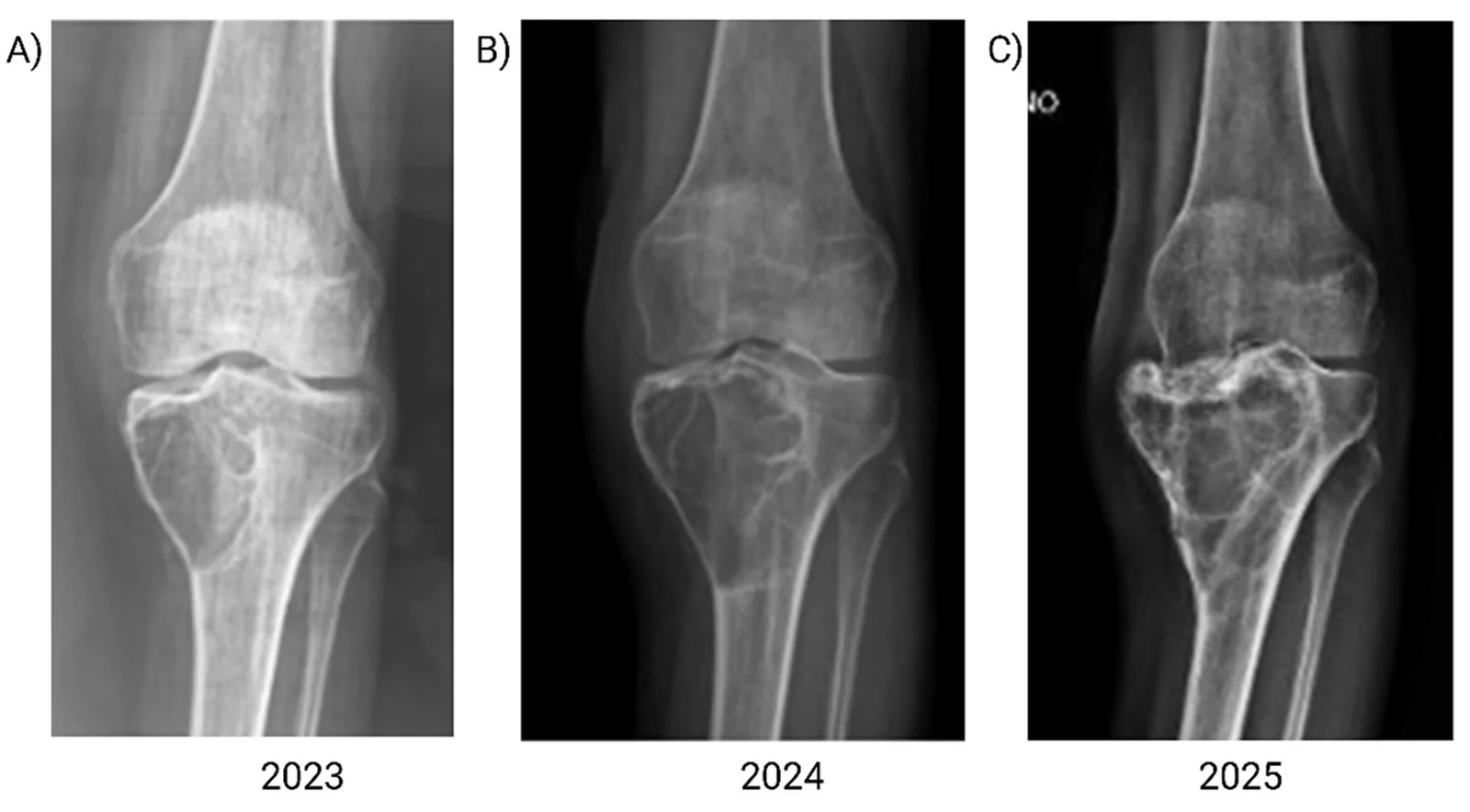
Radiographic evolution of giant cell tumor of the left proximal tibia. **(A–C)** Sequential anteroposterior radiographs obtained in 2023, 2024, and 2025 showing progressive expansion of the proximal tibial lytic lesion, cortical destruction, and articular collapse. Increased sclerosis and peripheral neo-osteogenesis are observed in the latest preoperative image after denosumab treatment.

Further diagnostic workup included bone scintigraphy, computed tomography (CT), and magnetic resonance imaging (MRI). CT characterized the lesion as a large expansile process involving approximately three-quarters of the proximal tibia and extending to the articular surface (Figures 2A,B). MRI confirmed intramedullary involvement without gross invasion of the surrounding soft tissues (Figures 2C,D). In the absence of clear extraosseous soft-tissue extension, the lesion was radiographically categorized as Campanacci grade II, although marked subchondral collapse and structural compromise were already present (13).

**Figure 2:**
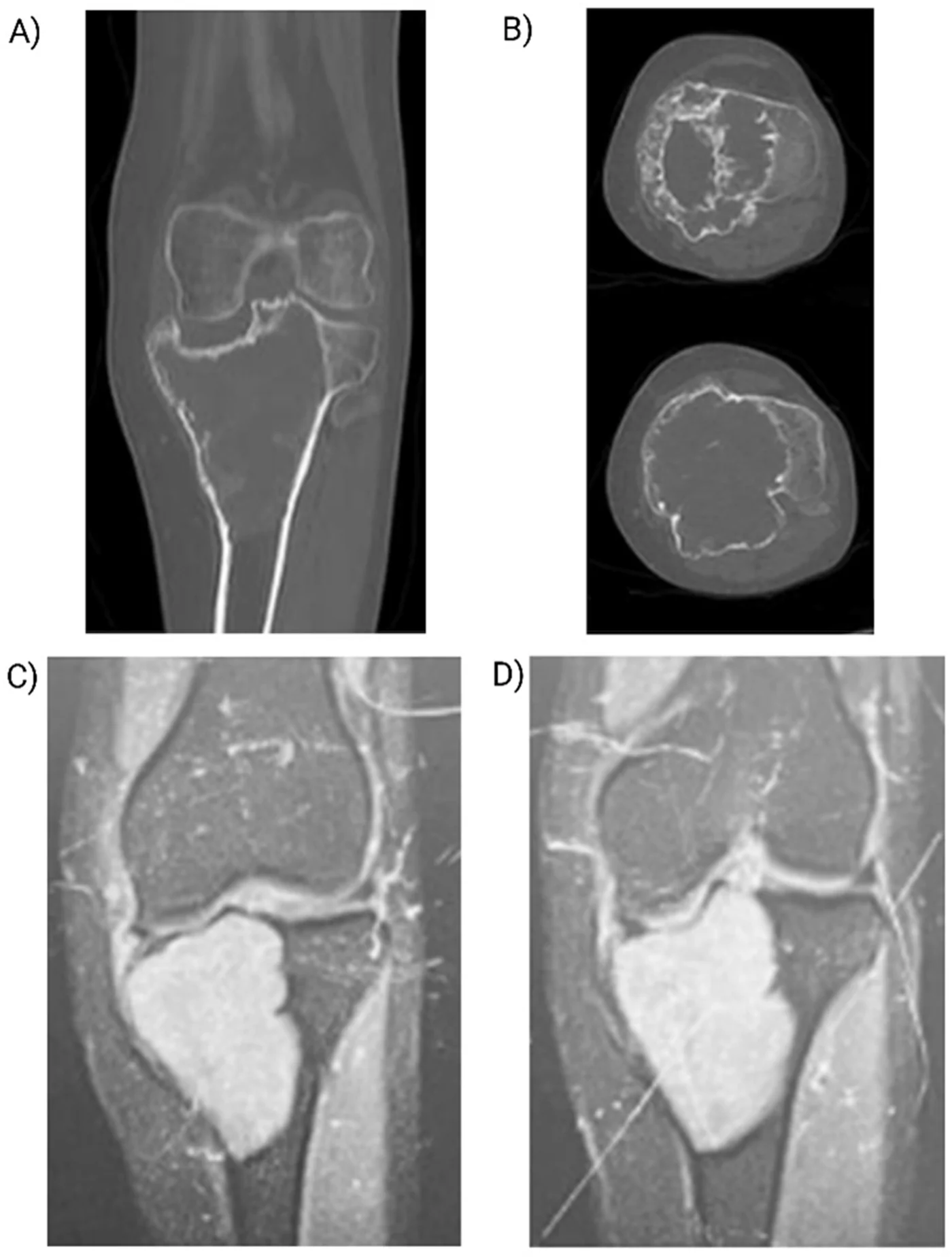
Preoperative cross-sectional imaging of the proximal tibial lesion. **(A)** Coronal CT and **(B)** axial CT demonstrate the extent of the expansile lesion and associated articular collapse. **(C,D)** MRI sequences show heterogeneous medullary involvement with subchondral extension and marked compromise of the joint surface.

Given the clinical and imaging findings, a percutaneous biopsy was performed in the operating room and confirmed the diagnosis of giant cell tumor of bone. The diagnosis was established on the basis of the official histopathological report from the biopsy specimen. Histopathological microphotographs were not available for inclusion because the pathology laboratory provided only the written diagnostic report. After multidisciplinary discussion, the lesion was considered unsuitable for intralesional reconstruction because of the extent of epiphyseal destruction, articular collapse, and anticipated mechanical insufficiency. The patient was therefore scheduled for oncologic resection of the proximal tibia using patient-specific cutting guides, followed by reconstruction with a custom 3D-printed implant and hinged total knee arthroplasty. During the interval required for implant manufacturing, the patient received six cycles of denosumab, every four weeks at a dose of 120 mg subcutaneously which leads to slowing of osteoclastic proliferation and accelerates the formation of mature rim around the tumor. After the commercial introduction of denosumab, a marked reduction in time of resection and reconstruction was observed; The osteosclerotic rim that is formed use of denosumab, allows the surgeon to conveniently identify the tumor margins and proceed easier and narrower resections with lesser chances of tumor spillage (14).

As part of the preoperative planning, patient-specific cutting guides were designed using 3D technology to match the anatomy of the residual tibia while preserving a planned 10-mm oncologic margin in the remaining bone (Figure 3A). For 3D reconstruction, the software Mimics Medical was used, and for CAD modeling, the software 3-matic, both developed by Materialise, Belgium. In parallel, a custom proximal tibial implant with a porous trabecular architecture was developed to fit the defect and support the tibial component of the hinged knee prosthesis (Figure 3B). The implant incorporated three fixation eyelets on its anterior and proximal surfaces to facilitate reinsertion of the extensor mechanism. It also featured a hollow endomedullary stem measuring 56 mm in length, with a total height of 105 mm, excluding the epiphyseal replacement designed to receive the tibial component of the prosthesis. Additional fixation was planned using two anterior and two oblique screws measuring 12 to 14 mm. The implant was specifically designed to accommodate a GKS Butterfly® hinged knee prosthesis with the following components: tibia, small; femur, small; insert, 10. The analog planning was carried out by the Department of Oncotraumatology of the hospital. Although the lesion was classified as Campanacci grade II, an en bloc resection was performed due to its articular location. The literature recommends a margin between 10 and 20 mm of healthy tissue to ensure the absence of neoplastic cells at the margin (R0 margin). Therefore, the resection was planned at 1 cm below the lesion, transferring this measurement to the cutting guide, which was positioned proximally over the lesion

**Figure 3:**
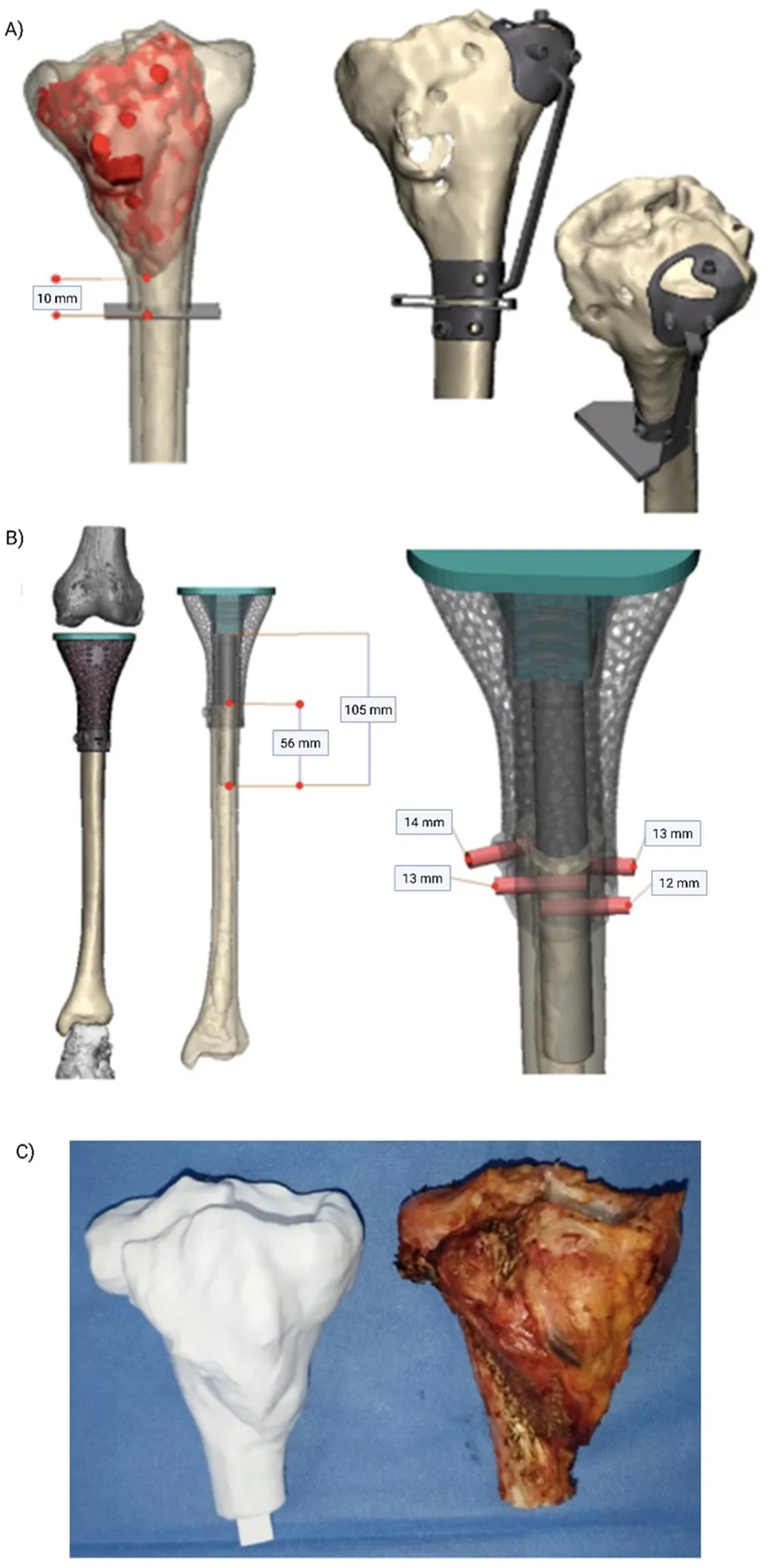
Three-dimensional preoperative planning and implant design. **(A)** Tumor mapping, planned osteotomy, oncologic margins, and patient-specific cutting guide. **(B)** Custom-made proximal tibial osteoarticular implant designed to fit the residual tibia and support the tibial component of the hinged knee prosthesis. **(C)** Comparison between the three-dimensional printed anatomical model and the resected specimen.

An extended anteromedial approach to the knee was performed. After posterior release of the medial compartment and the pes anserinus, the medial gastrocnemius muscle was identified and mobilized. The extensor mechanism was detached for later reconstruction, and the joint was exposed together with the tumoral lesion (Figures 4A–C). Once adequate exposure had been achieved, the collateral ligaments were sectioned, allowing complete mobilization of the proximal tibia. The popliteal neurovascular bundle was carefully protected throughout the procedure.

**Figure 4:**
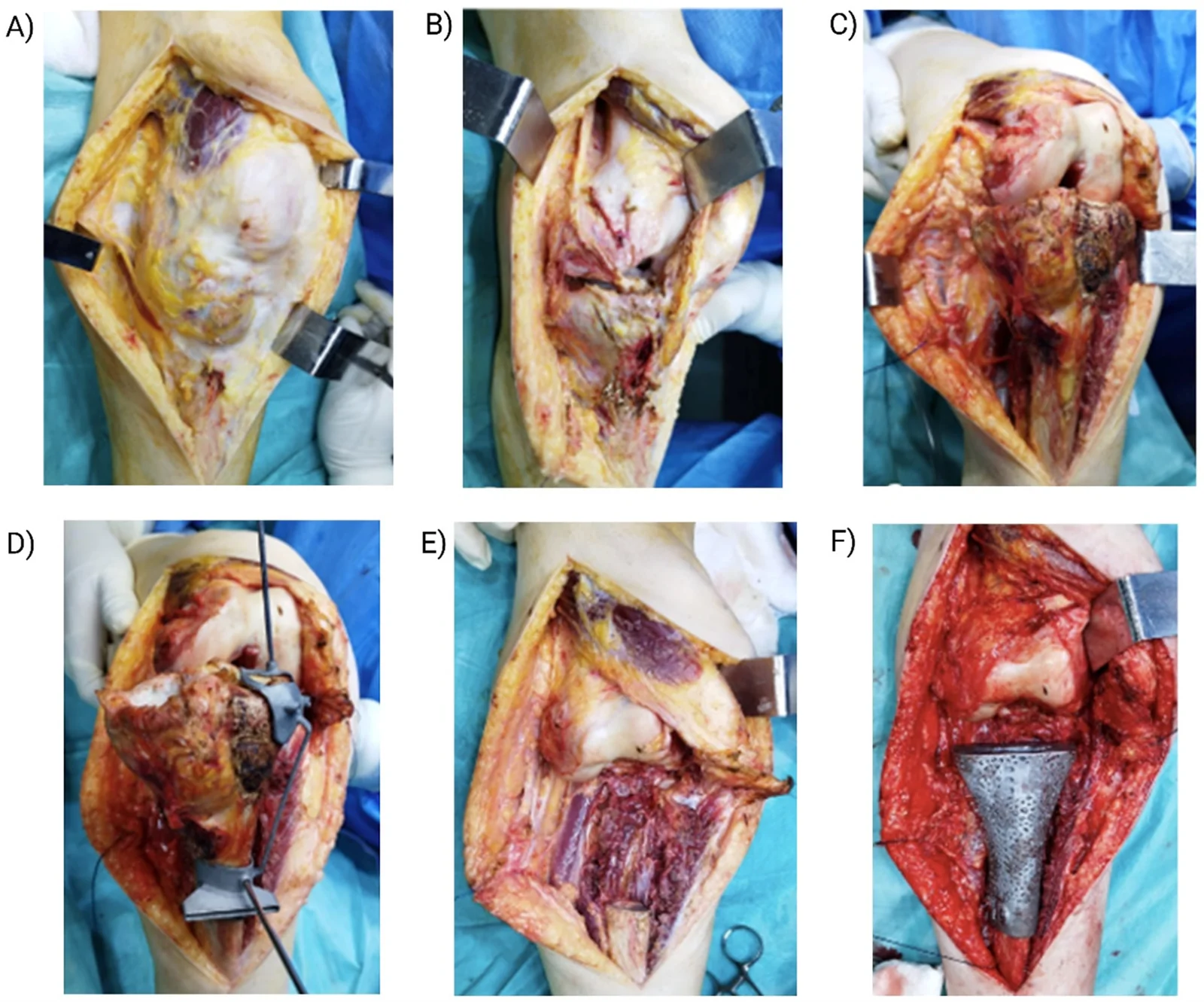
Intraoperative sequence. **(A)** Extended anteromedial approach to the knee. **(B)** Articular exposure. **(C)** Tumor dissection and release. **(D)** Positioning of the cutting guide. **(E)** Resection of the proximal tibia. **(F)** Trial and definitive positioning of the custom implant.

The patient-specific cutting guide was then positioned over the lesion, and the osteotomy was performed with an oscillating saw (Figures 4D,E). Following en bloc resection of the proximal tibia, the custom implant was inserted by press-fit, achieving intimate contact at the implant–bone interface (Figure 4F). The arthroplasty phase was then initiated. Cutting guides were placed on the distal femur, the corresponding bone cuts were performed, and the trial components were tested. Finally, the definitive components were cemented, and a constrained hinged prosthesis with a + 1 tibial step was implanted.

After completion of the arthroplasty phase, the extensor mechanism was reinserted through the fixation eyelets incorporated into the tibial implant, with the knee maintained in flexion. Soft-tissue coverage was achieved using a medial gastrocnemius flap combined with the pes anserinus and polypropylene mesh reinforcement (Figure 5). Final implant position and stability were confirmed intraoperatively using fluoroscopy.

**Figure 5:**
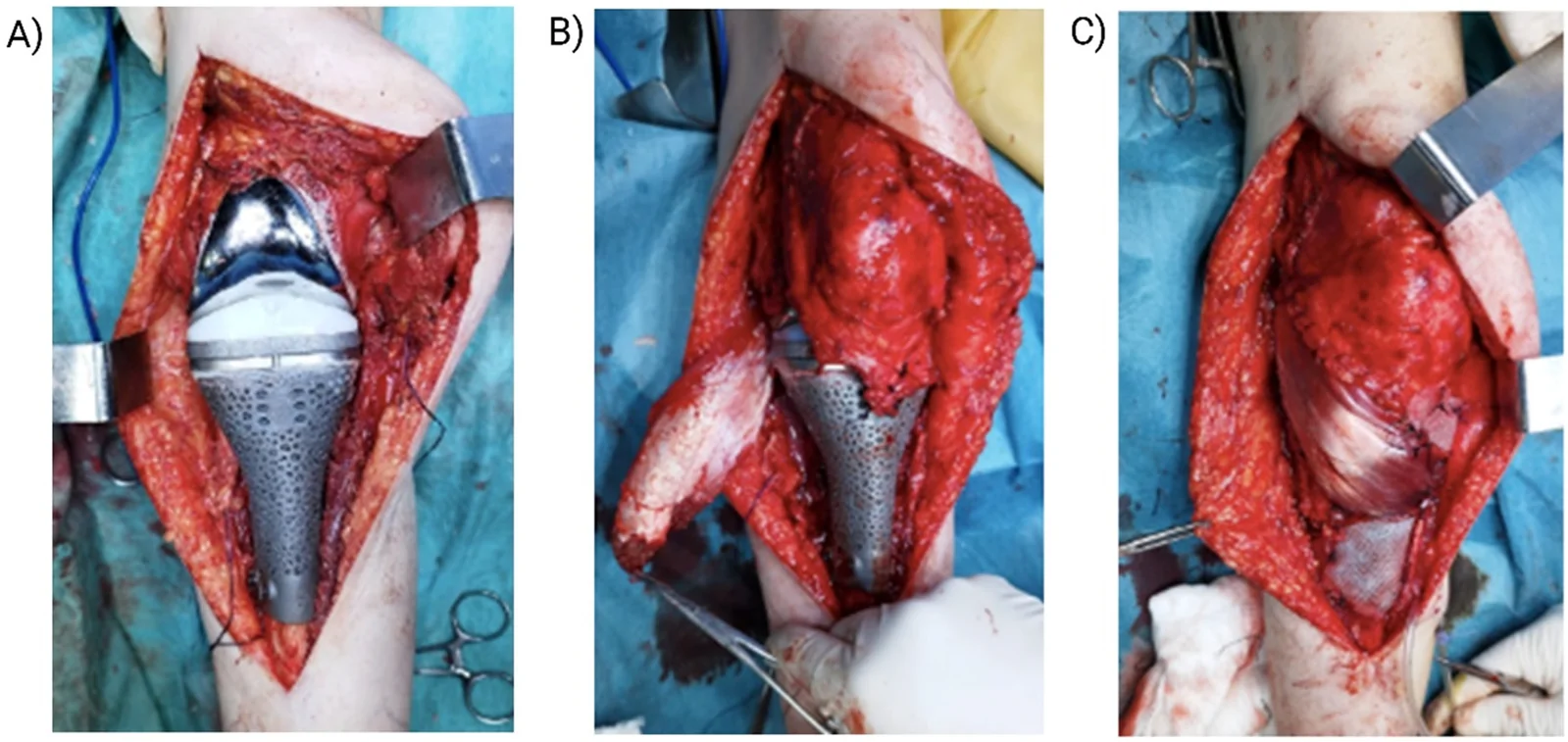
Final reconstructive steps. **(A)** Placement of the cemented hinged knee prosthesis. **(B)** Reinsertion of the extensor mechanism. **(C)** Medial gastrocnemius flap coverage and soft-tissue reconstruction.

The postoperative wound healing process was uneventful, with no clinical signs of infection, wound dehiscence, or early mechanical complications. Progressive mobilization was initiated on postoperative day 10, followed by supervised physiotherapy focused on quadriceps strengthening and preservation of knee range of motion. At 6 weeks, the patient achieved full weight-bearing with the assistance of a walker, and by 4 months she was able to ambulate using a cane.

Follow-up radiographs obtained at 6 months demonstrated satisfactory alignment of the construct and radiographic evidence consistent with early structural integration, with no signs of local recurrence, implant loosening, or delayed healing (Figures 6A,B). Based on these findings, unassisted ambulation was recommended. At the latest evaluation, the patient reported no pain or gait claudication, achieved an active knee range of motion from 10° to 90° (80° arc), and tolerated full weight-bearing.

**Figure 6:**
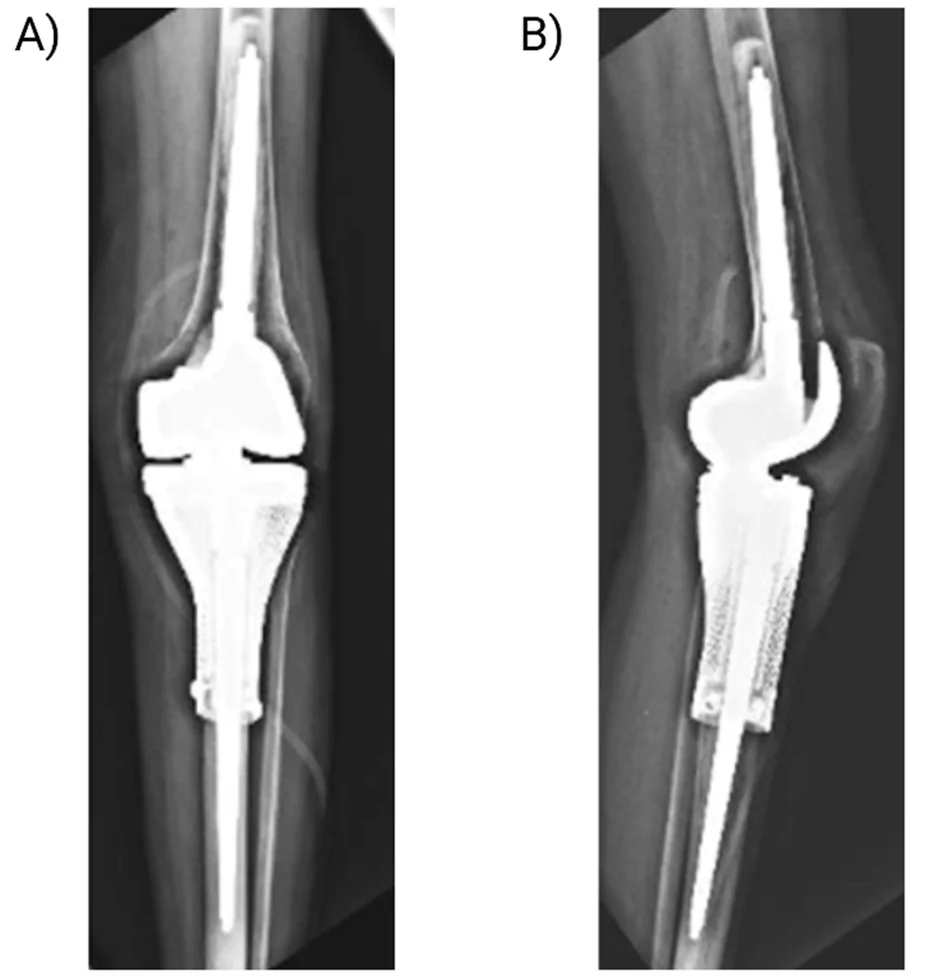
Postoperative anteroposterior **(A)** and lateral **(B)** radiographs demonstrating satisfactory alignment of the custom proximal tibial implant and cemented hinged knee prosthesis, with no radiographic evidence of loosening or early mechanical failure.

Objective functional assessment was performed using the Musculoskeletal Tumor Society (MSTS) score, which evaluates pain, function, emotional acceptance, use of supports, walking ability, and gait pattern on a scale from 0 to 30, with higher scores indicating better function (15). At the 6-month follow-up, the patient's MSTS score improved from 19 preoperatively to 30, reflecting an excellent functional outcome (16).

As this is a reconstructive procedure, requiring meticulous planning, proper positioning, and, most importantly, an optimal press-fit of the implant onto the remaining tibia, the expected implant survival is high. This is because the goal is not only mechanical stability but also biological fixation, achieved through the material's porosity compared to traditional methods. Therefore, despite the longer surgical time and higher material cost, we consider custom-designed trabecular metal implants to be a viable option in cases of extensive bone defects following oncologic resections, as in our patient's case.

## 3 Discussion

The surgical management of GCTB remains influenced by tumor grade, structural compromise, and anatomical location. In general, Campanacci grade I and II lesions are often managed with intralesional curettage, high-speed burring, and defect filling with bone graft or cement, whereas grade III lesions with cortical destruction and soft-tissue extension more commonly require en bloc resection and reconstruction when needed (16). However, radiographic grade alone may not fully capture the degree of mechanical non-reconstructability in periarticular lesions (17).

In parallel, the surgical staging principles described by Enneking integrate the biological behavior and anatomical extent of the lesion to guide margin selection (18, 19). Benign aggressive tumors with cortical destruction, progressive collapse, or severe articular compromise may justify wide excision when limb-salvage reconstruction is technically feasible (18, 19). In the present case, although the lesion was categorized as Campanacci grade II because no gross soft-tissue extension was identified, it involved approximately 75% of the proximal tibial epiphysis and was associated with marked articular collapse and biomechanical compromise. Under these circumstances, an intralesional procedure would have offered limited potential for durable joint preservation and may have carried an increased risk of recurrence and mechanical failure. Indeed, recurrence after intralesional curettage in structurally compromised lesions has been reported in the range of 10%–35%, whereas wide resection may reduce recurrence to 0%–5% in selected patients (4, 16).

Reconstruction of the proximal tibia following wide tumor resection presents unique biomechanical and soft-tissue challenges. The procedure must restore axial load transmission, joint alignment, and continuity of the extensor mechanism in a region with limited soft-tissue coverage and close proximity to major neurovascular structures (6, 19).

The problems associated with proximal tibial reconstructions are related to the relatively limited soft-tissue coverage of the wound and the unreliable options available for reconstruction of the extensor mechanism (20). Conventional megaprostheses provide immediate stability and allow early weight-bearing, but they are associated with complications such as aseptic loosening, extensor mechanism insufficiency, and implant failure. Henderson et al. reported mechanical complication rates ranging from 15% to 30% in tumor endoprostheses (21). Additionally, it has been described that patients with metallic endoprostheses have lower rates of complications and amputation, as well as higher survival rates, compared with those treated with allograft reconstructions (22)**.**

Allograft–prosthesis composite reconstruction may restore bone stock, but it also carries substantial risks, including nonunion, graft fracture, infection, and late structural collapse, with complication rates reaching 30%–40% in long-term series (23). In contrast, cement reconstruction following curettage remains more appropriate for contained lesions and may be insufficient when there is extensive epiphyseal destruction and collapse of the joint surface (4, 16).

In this context, custom-made porous implants appear to be a viable option, offering advantages such as anatomical customization tailored to the defect and the potential for biological fixation. A recent systematic review evaluated patients treated with custom-made trabecular metal implants for segmental bone defects after wide resection and reported radiographic osseointegration in most cases at 6 months, supporting the biological rationale of this strategy (24). In addition, porous architectures ranging from 400 to 600 μm have been associated with bone ingrowth and improved implant–bone interface stability, which may be especially advantageous in metaphyseal reconstructions exposed to high mechanical demands (24, 25). In the present case the trabecular metal implant is a highly porous titanium conformation of the Ti6Al4V-ELI type; in the form of an interconnected matrix, permeable to bone tissue growth and vascularization. It has an elastic modulus of 5,000 to 15,000 MPa for cortical bone, 2 mm cells, and 1 mm pores, manufactured using laser additive manufacturing (SLM) with a Renishaw AM400 system.

The implant was specifically tailored to the residual tibia, incorporated fixation features for extensor mechanism reinsertion, and allowed supplementary screw fixation in addition to press-fit stability and the cemented hinged prosthetic reconstruction (24).

In a retrospective cohort study of extremity bone tumor reconstruction, customized 3D-printed prostheses were associated with shorter operative time than conventional allograft reconstruction, supporting the possibility of workflow-related efficiency gains, However, it should be emphasized that cohorts with longer follow-up periods are needed to evaluate clinical efficacy (26). In our patient's case, total operative time was 4.5 hours, which falls within the range reported for complex custom reconstructions and reflects the added steps required for oncologic resection, implant fitting, and extensor mechanism reconstruction (26, 27). In addition, patient-specific disposable instrumentation has been associated with reduced instrument-tray burden in knee arthroplasty, which may translate into logistical and sterilization-related savings (28). Nevertheless, the economic impact of patient-specific workflows remains highly context-dependent and was not formally quantified in our institution; therefore, cost considerations in this case should be interpreted qualitatively rather than as a formal cost-effectiveness analysis.

Another strength of this approach was the use of virtual planning and patient-specific cutting guides. These tools improved preoperative understanding of the lesion, facilitated accurate osteotomy planning, and helped translate the planned margins into the operating room (Figure 3C). In anatomically complex reconstructions, this level of personalization may reduce intraoperative uncertainty and improve construct fit, both of which are especially valuable in orthopedic oncology.

Despite the favorable short-term outcome, this report has important limitations. First, as this is a single case, the findings and results cannot be generalized to a broader patient population. These results also cannot be interpreted as evidence of the superiority of personalized reconstruction using 3D printing over established reconstructive techniques. In addition, due to the absence of a control group, a meaningful comparative evaluation of clinical outcomes, complications, implant survival, or functional recovery cannot be performed. It is also important to note that, although the 6-month follow-up period showed favorable results, it is insufficient to assess long-term oncological control or the mechanical durability of the reconstruction, as some complications may occur at a later stage and therefore cannot be adequately evaluated in the present case. Similarly, the durability of the functional improvement observed during the initial follow-up remains uncertain. Therefore, further studies with larger cohorts, longer follow-up periods, and, preferably, comparative designs are necessary to determine the reproducibility and true clinical value of this approach.

## 4 Conclusion

The integration of virtual planning, patient-specific cutting guides, and a custom porous trabecular implant combined with hinged knee arthroplasty appears to be a feasible limb-salvage strategy for selected patients with proximal tibial GCTB and major articular destruction. In this case, the construct provided early mechanical stability, enabled extensor mechanism reconstruction, and showed radiographic evidence of osseointegration without recurrence or loosening at 6 months. Although longer follow-up is necessary, this approach may represent a valuable alternative when conventional intralesional or standard reconstructive options are unlikely to restore both oncologic safety and knee function.

## Data Availability

The original contributions presented in the study are included in the article/Supplementary Material, further inquiries can be directed to the corresponding author.
